# Type of atrial fibrillation and outcomes in patients without oral anticoagulants

**DOI:** 10.1002/clc.23519

**Published:** 2020-12-12

**Authors:** Jiameng Ren, Yanmin Yang, Jun Zhu, Shuang Wu, Juan Wang, Han Zhang, Xinghui Shao, Siqi Lyu

**Affiliations:** ^1^ Emergency and Intensive Care Center, State Key Laboratory of Cardiovascular Disease Fuwai Hospital, National Center for Cardiovascular Diseases, Chinese Academy of Medical Sciences and Peking Union Medical College Beijing China

**Keywords:** outcomes, paroxysmal atrial fibrillation, persistent or permanent atrial fibrillation, risk predictor, type of atrial fibrillation

## Abstract

**Background:**

The effect of type of atrial fibrillation (AF) on adverse outcomes in Chinese patients without oral anticoagulants (OAC) was controversial.

**Hypothesis:**

The type of AF associated with adverse outcomes in Chinese patients without OAC.

**Methods:**

A total of 1358 AF patients without OAC from a multicenter, prospective, observational study was included for analysis. Univariable and multivariable Cox regression models were utilized. Net reclassification improvement analysis was performed for the assessment of risk prediction models.

**Results:**

There were 896(66%) patients enrolled with non‐paroxysmal AF (NPAF) and 462(34%) with paroxysmal AF (PAF). The median age was 70.9 ± 12.6 years, and 682 patients (50.2%) were female. During 1 year of follow‐up, 215(16.4%) patients died, and 107 (8.1%) patients experienced thromboembolic events. Compared with the PAF group, NPAF group had a notably higher incidence of all‐cause mortality (20.2% vs. 9.4%, *p* < .001), thromboembolism (10.5% vs. 3.8%, *p <* .001). After multivariable adjustment, NPAF was a strong predictor of thromboembolism (HR 2.594, 95%CI 1.534–4.386; *p <* .001), all‐cause death (HR 1.648, 95%CI 1.153–2.355; *p* = .006). Net reclassification improvement analysis indicated that the addition of NPAF to the CHA_2_DS_2_‐VASc score allowed an improvement of 0.37 in risk prediction for thromboembolic events (95% CI 0.21–0.53; *p <* .001).

**Conclusions:**

In Chinese AF patients who were not on OAC, NPAF was an independent predictor of thromboembolism and mortality. The addition of NPAF to the CHA_2_DS_2_‐VASc score allowed an improvement in the accuracy of the prediction of thromboembolic events.

## 1 INTRODUCTION

In 2020 ESC Guidelines for the diagnosis and management of atrial fibrillation, the “4S‐AF” scheme^1^ was proposed to be considered in managing atrial fibrillation (AF) patients. The burden of AF has been listed as one component, and studies demonstrated that the duration or pattern of AF correlates with the extent of the atrial substrate, remodeling, and AF‐related outcomes.^1^ The AF pattern is now the simplest and quick way to assess patients' AF burden without much examination. Though the effects of AF pattern on outcomes had been investigated for nearly 20 years, the results were controversial. Most of these papers enrolled patients with oral anticoagulants (OACs), and anticoagulant state may also be a confounder. As so far, only two studies^2^, ^3^ focused the non‐anticoagulated patients, and the results were not consistent. More information is needed to assess the prognosis of AF type on adverse events in patients without OAC. Though China has a heavy burden of AF that the annual risk of thromboembolic events in Chinese AF patients ranged from 3.7% to 9.2%,^4^, ^5^, ^6^ there was little data about the relationship between AF pattern and outcomes. The purpose of this article was to explore the association between AF type and adverse outcomes in Chinese patients who were not on anticoagulation and further evaluated the value of AF pattern in decision‐making for stroke prevention.

## 2 METHODS

The present study was based on a multicenter, prospective, observational study^7^ in China with 1‐year follow‐up, in which 2016 patients with AF were enrolled consecutively at the emergency department from November 2008 to October 2011. The diagnosis of AF was confirmed by reviewing clinical records, electrocardiographic evidence, and electronic databases according to International Classification of Disease, 9th Revision, Clinical Modification Diagnostic Code 427.31 or 427.32. Twenty representative hospitals around China (including rural and urban, academic and community, general and specialized, public and private hospitals) had participated. A total of 1358 patients with non‐valvular AF and no OAC (both discharged without OAC and no initiation during the follow‐up) from the multicenter study were included for analysis in the present article. The process of patient selection was shown in Figure S1. The low percentage of anticoagulation therapy in our study was a national medical status, and the proportion was only 2.7% in 2004^8^ and increased to 18.6% ~ 31.7% in the early period of the 2010s.^7^, ^9^ The study was approved by the ethics committee of each center and obeyed the Declaration of Helsinki. All patients have provided written consent to participate in the study.

The demographic information, admission vital signs, medical histories, and treatments were collected at baseline by interviewing the participants, reviewing their medical records, and contacting their treating physicians. The type of AF was defined according to the 2006 ACC/AHA/ESC guidelines for the management of patients with AF.^10^ Briefly, if the arrhythmia terminated spontaneously, AF was designated paroxysmal; when sustained beyond 7 days, it was termed persistent. Termination with pharmacological therapy or direct‐current cardioversion does not alter the designation. Permanent AF was defined that AF did not terminate either spontaneously or with electrical or chemical cardioversion, or cardioversion had not been attempted. Both persistent AF and permanent AF were divided into non‐paroxysmal AF (NPAF) group in the following analysis. There were 896 (66%) patients enrolled with NPAF and 462 (34%) with paroxysmal AF (PAF). The classification of the subtype of AF relied on the attending physician's interpretation. Patients were also divided into AF/flutter group or other rhythm group by the electrocardiogram rhythm at discharge for sensitivity analysis. The CHA_2_DS_2_‐VASc score by giving 2 points to each patient of age ≥75 years and a history of prior stroke or TIA and 1 point to each patient of age 65–74 years, history of hypertension, diabetes mellitus, congestive heart failure, vascular diseases, and female sex.

The registry was designed to have a 1‐year follow‐up. The follow‐up was completed in November 2012 by trained research personnel via clinic visit, telephone or delivery of medical records. The status of OAC during the follow‐up period was collected again at the visit. In this study, the primary outcome was thromboembolic events (TE events, including stroke and non‐central nervous system embolism), and secondary outcomes were defined as all‐cause death, cardiovascular death and stroke. Cardiovascular death included sudden cardiac death and death caused by heart failure, stroke, myocardial infarction, pulmonary embolus, peripheral embolus, aortic dissection.

Continuous variables were expressed as means with *SD*s or medians with quartiles; categorical variables were expressed as frequencies and percentages. Differences in continuous variables between groups according to the type of AF were analyzed using unpaired *t*‐test or the Mann–Whitney U test; comparison of categorical variables was performed using *χ* ^2^ test or Fisher's exact test. Kaplan–Meier curves and log‐rank tests were performed to illustrate the discrepancies among the AF patterns. Univariable and multivariable Cox regression analysis was utilized to evaluate the effects of AF type on the TE events, stroke, all‐cause death and cardiovascular death. The following covariables were adjusted in the multivariable model: sex, age ≥75‐years‐old, body mass index, admission systolic blood pressure, admission diastolic blood pressure, admission heart rate, tobacco use, previous stroke or transient ischemic attack (TIA), coronary artery disease (CAD), prior myocardial infarction, hypertension, HF, significant valvular heart disease, diabetes mellitus, emphysema/chronic obstructive pulmonary disease, hyperthyroidism, sleep apnea, previous major bleeding, dementia or cognitive defects, antiplatelet drug, β‐blocker, ACEI/ARB, calcium channel blocker, diuretics, digoxin, statin, antiarrhythmic drug. Further, we conducted subgroup analyses to assess whether the difference between types on the risk of TE events and all‐cause death existed among the following specific subsets of patients, including sex, age, presence of CAD, hypertension, HF, CHA_2_DS_2_‐VASc score and antiplatelet drugs at discharge. Hazard ratio was estimated using Cox proportional hazards models fitted separately in subgroups of patients. The association between the rhythm at discharge (AF/flutter group vs other rhythm group) and adverse outcomes were also explored as the sensitivity analysis. The incremental contribution of AF type in predicting the risk of TE events based on CHA_2_DS_2_‐VASc score was presented as net reclassification improvement and integrated discrimination improvement using the PredicABEL, an R package for the assessment of risk prediction models. The prognostic utility of CHA_2_DS_2_‐VASc score and after adding NPAF as a risk factor (1 point) into CHA_2_DS_2_‐VASc score was assessed by C‐statistic estimates. The comparison between the two scores was also made.

Hazard ratio and 95% confidence intervals (CI) were calculated. The software package SPSS version 25.0 (IBM Corporation, New York, NY), the R software version 4.0.2 (R Foundation for Statistical Computing) and the MedCalc version 19.0.7 were used for statistical analysis. GraphPad Prism version 6.01 was utilized for figures. All statistical tests were two‐tailed, and a *p*‐value <.05 were considered significant.

## 3 RESULTS

Baseline characteristics were given in Table 1. Compared to 462 (34%) patients admitted with PAF, patients with NPAF were older and had higher systolic blood pressure, CHA_2_DS_2_‐VASc score, but lower body mass index, diastolic blood pressure, and heart rate. They suffered from comorbidities more frequently. Patients with NPAF were more likely to take antiplatelet drugs, diuretics and digoxin, while they had less proportion of antiarrhythmic agents. Baseline characteristics of patients classified by the rhythm at discharge were given in Table S1.

**TABLE 1:** Baseline characteristics of patients

Variables	Total	Paroxysmal AF	Persistent or permanent AF	*p*‐value
	*n* = 1358	*n* = 462, 34%	*n* = 896, 66%	
Demographics				
Female, *n*(%)	682 (50.2%)	237 (51.3%)	445 (49.7%)	.568
Age ≥ 75 years, *n*(%)	613 (45.1%)	169 (36.6%)	444 (49.6%)	<.001
BMI, kg/m^2^	23.8 ± 3.7	24.2 ± 3.4	23.6 ± 3.9	.01
SBP, mmHg	130 (120–150)	130 (115.8–145)	135 (120–150)	<.001
DBP, mmHg	80 (70–90)	80 (70–89)	80 (70–90)	.031
HR, bpm	100 (80–123)	101.9 (81–130)	98 (80–120)	.002
CHA_2_DS_2_‐VAS score	3.7 ± 2.0	3.1 ± 1.9	4.0 ± 2.0	<.001
Tobacco use, *n*(%)	320 (23.6%)	103 (22.3%)	217 (24.2%)	.429
Medical history, *n*(%)				
Hypertension	846 (62.3%)	283 (61.3%)	563 (62.8%)	.569
Heart failure	463 (34.1%)	92 (19.9%)	371 (41.4%)	<.001
Coronary artery disease	683 (50.3%)	183 (39.6%)	500 (55.8%)	<.001
Previous myocardial infarction	116 (8.5%)	35 (7.6%)	81 (9%)	.36
Previous stroke or TIA	256 (18.9%)	57 (12.3%)	199 (22.2%)	<.001
Diabetes mellitus	232 (17.1%)	70 (15.2%)	162 (18.1%)	.174
Significant valvular heart disease	46 (3.4%)	6 (1.3%)	40 (4.5%)	.002
Emphysema/COPD	180 (13.3%)	41 (8.9%)	139 (15.5%)	.001
Hyperthyroidism	53 (3.9%)	16 (3.5%)	37 (4.1%)	.548
Sleep apnea	54 (4%)	19 (4.1%)	35 (3.9%)	.854
Major bleeding	33 (2.4%)	7 (1.5%)	26 (2.9%)	.116
Dementia or cognitive defects	33 (2.4%)	8 (1.7%)	25 (2.8%)	.23
AF at discharge	983 (72.4%)	193 (41.8%)	790 (88.2%)	<.001
Prior AF catheter, surgical ablation or Maze procedure	11 (0.8%)	8 (1.7%)	3 (0.3%)	.01
Medication at discharge, *n*(%)				
Antiplatelet drug	868 (63.9%)	278 (60.2%)	590 (65.8%)	.039
β‐blocker	595 (43.8%)	199 (43.1%)	396 (44.2%)	.693
ACEI/ARB	541 (39.8%)	168 (36.4%)	373 (41.6%)	.06
Calcium channel blocker	368 (27.1%)	140 (30.3%)	228 (25.4%)	.056
Diuretics	442 (32.5%)	87 (18.8%)	355 (39.6%)	<.001
Digoxin	302 (22.2%)	54 (11.7%)	248 (27.7%)	<.001
Statin	402 (29.6%)	130 (28.1%)	272 (30.4%)	.396
Antiarrhythmic drug	164 (12.1%)	91 (19.7%)	73 (8.1%)	<.001

Abbreviations: ACEI/ARB: angiotensin‐converting enzyme inhibitor/angiotensin receptor antagonist; AF, atrial fibrillation; BMI, body mass index (calculated by dividing weight in kilograms by the square of height in meters); COPD, chronic obstructive pulmonary disease; DBP, diastolic blood pressure; HR, heart rate; SBP, systolic blood pressure; TIA, transient ischemic attack.

**FIGURE 1:**
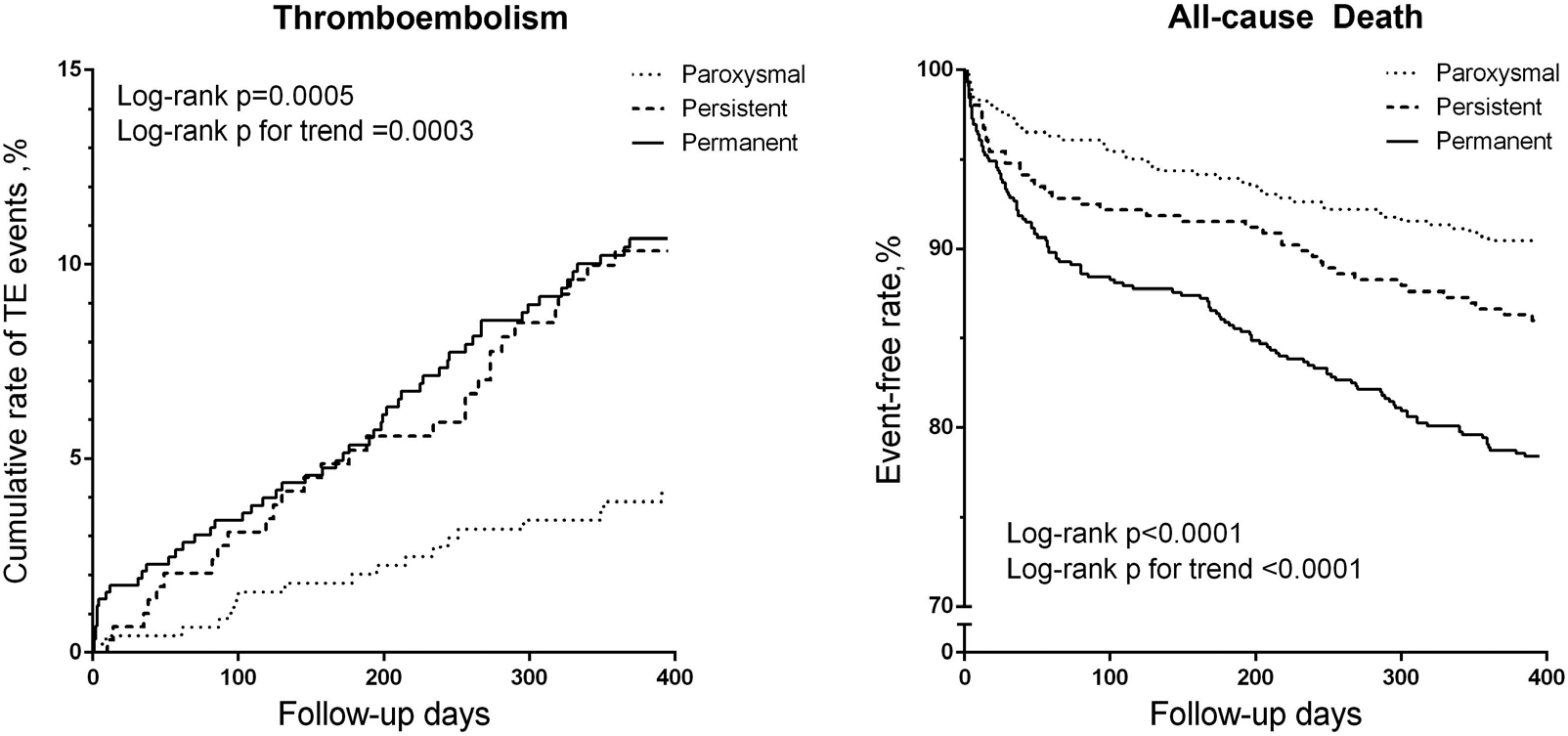
Kaplan–Meier curves of cumulative rate of thromboembolic events and survival according to the type of atrial fibrillation

**FIGURE 2:**
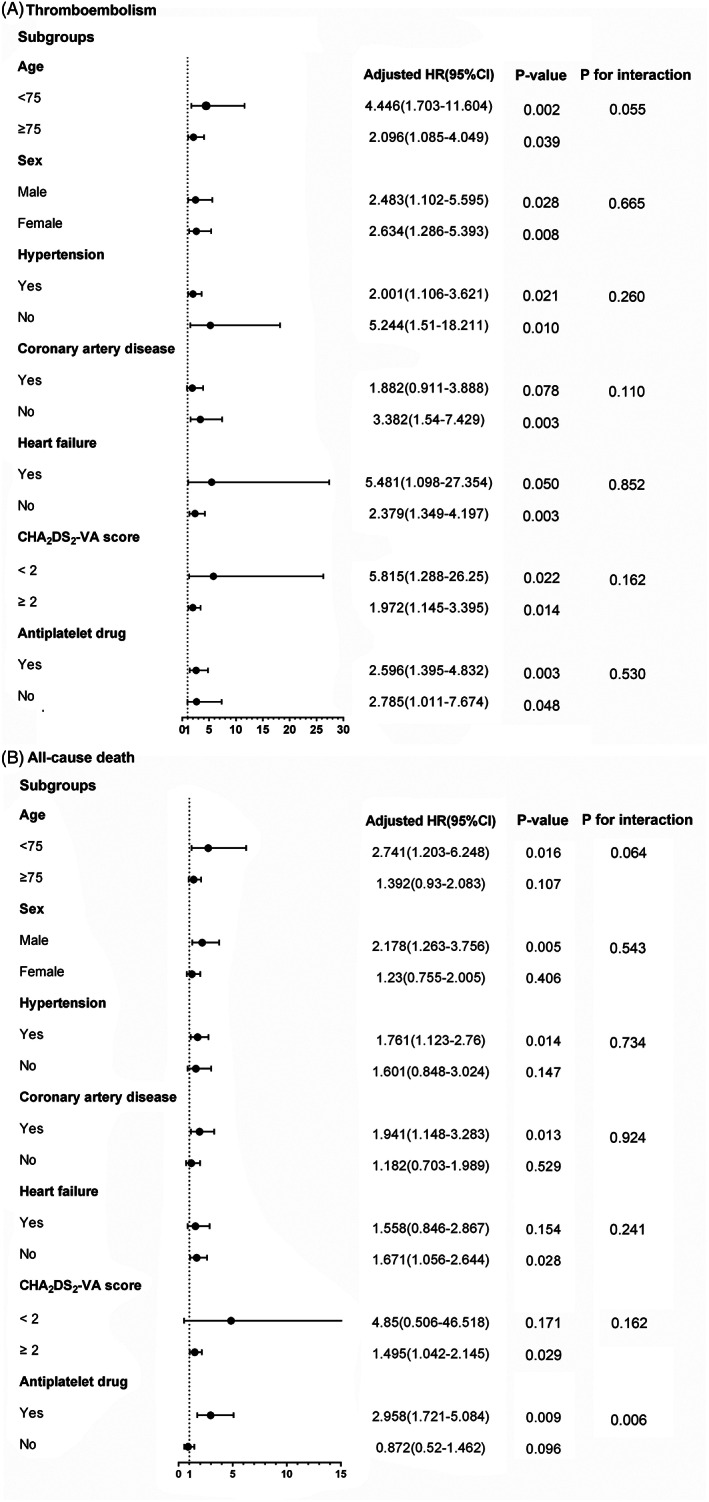
The risk of thromboembolism and all‐cause death in subgroups. (paroxysmal atrial fibrillation as the reference). CI, confidence interval; HR, hazard ratio

**FIGURE 3:**
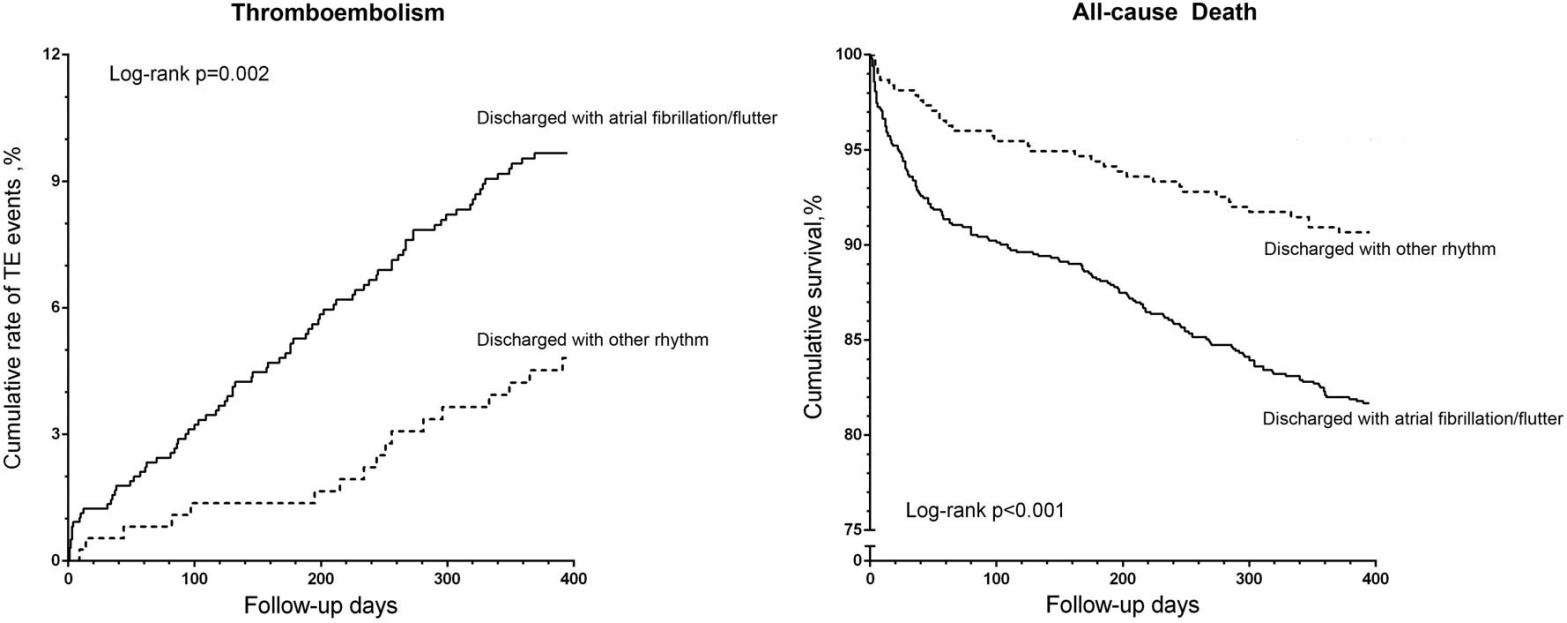
Kaplan–Meier curves of cumulative rate of thromboembolic events and survival according to the rhythm at discharge

The adverse outcomes during the 1314 person‐year of follow‐up and the relationship with AF type were given in Table 2. Kaplan–Meier curves of the cumulative rate of TE events and cumulative survival according to AF types were illustrated in Figure 1. After adjusting for the risk factors in CHA_2_DS_2_‐VASc score and other confounders, NPAF was still a strong independent predictor of thromboembolism, stroke, and all‐cause death and the tendency remained for cardiovascular death. The prognostic value of AF patterns in the subgroups was also assessed **(**Figure 2 **)**. As for the TE events, the results were consistent in all subgroups. Regarding the all‐cause death, the trend existed in all subgroups except for the patients discharged without antiplatelet agents (p for interaction = 0.006). Furthermore, sensitivity analysis suggested that the main findings were consistent (Figure 3, Table S2). Other baseline risk factors of TE events in this population were age ≥75‐years‐old, female and prior stroke or TIA (Table S3).

**TABLE 2:** The risk of outcomes in patients with NPAF comparing to PAF

	Numbers of events (yearly rate, %)			Univariable analysis		Multivariable analysis^a^	
Outcomes	All patients	Patients with PAF	Patients with NPAF	HR (95%CI)	*p*‐value	HR (95%CI)	*p*‐value
Thromboembolism	107 (8.1)	18 (3.8)	89 (10.5)	2.802 (1.688–4.65)	<.001	2.594 (1.534–4.386)	<.001
Stroke	101 (7.7)	18 (3.8)	83 (9.8)	2.606 (1.565–4.338)	<.001	2.435 (1.434–4.134)	.001
All‐cause death	215 (16.4)	44 (9.4)	171 (20.2)	2.117 (1.52–2.948)	<.001	1.648 (1.153–2.355)	.006
Cardiovascular death	122 (9.3)	22 (4.7)	100 (11.8)	2.483 (1.565–3.94)	<.001	1.589 (0.963–2.623)	.07

Abbreviations: CI, confidence interval; HR, hazard ratio; NPAF, non‐paroxysmal atrial fibrillation; PAF, paroxysmal atrial fibrillation.

^a^ Adjusted for sex, age ≥75‐years‐old, body mass index, admission systolic blood pressure, admission diastolic blood pressure, admission heart rate, tobacco use, previous stroke or transient ischemic attack, coronary artery diseases, previous myocardial infarction, hypertension, heart failure, significant valvular heart disease, diabetes mellitus, emphysema/ chronic obstructive pulmonary disease, hyperthyroidism, sleep apnea, previous major bleeding, dementia or cognitive defects, antiplatelet drug, β‐blocker, ACEI/ARB, calcium channel blocker, diuretics, digoxin, statin, antiarrhythmic drug.

Besides, net reclassification improvement analysis indicated that the addition of NPAF to the CHA_2_DS_2_‐VASc score allowed an improvement of 0.37 in risk prediction for TE events (95% CI 0.21–0.53; *p <* .001). The integrated discrimination improvement was 0.34% (95% CI 0.19%–0.49%; *p <* .001). After adding NPAF into CHA_2_DS_2_‐VASc score, the c‐statistic (CHA_2_DS_2_‐VASc score as continuous variable) increased from 0.622 to 0.638 (z‐statistic 2.872, *p =* .0041).

## 4 DISCUSSION

Our data, from a multicenter, prospective, observational study, showed an increased risk of TE events in non‐anticoagulated patients with NPAF compared with those with PAF. After adjusting for variables, NPAF was still an independent predictor of thromboembolism and all‐cause death. The trend preserved when the AF pattern was classified into AF/flutter group or other rhythm group according to the electrocardiogram at discharge. The main findings were consistent in subgroups. Further, the addition of NPAF to the CHA_2_DS_2_‐VASc score allowed an improvement in the accuracy of the prediction of TE events.

Patients with NPAF had a higher arrhythmic burden than those with PAF. The AF pattern (first‐diagnosed, paroxysmal, persistent/long‐standing persistent, and permanent) is still the easiest manner to evaluate the burden in daily practice. The effect of the AF pattern on outcomes has been controversial for nearly two decades. Furthermore, there were few data in the Chinese population. In our study, the risk of all‐cause death increased from paroxysmal to persistent to permanent, which was in line with the results reported in The EORP‐AF General Pilot Registry.^11^ And patients with persistent or permanent AF had a similar risk of TE events. NPAF related to a 2.5‐fold increased risk of thromboembolism compared with PAF, which was higher than that in the pooled analysis of ACTIVE‐A and AVERROES.^2^ The baseline characteristics of patients were similar as previous studies reported, in which patients with NPAF had higher CHA_2_DS_2_‐VASc score and more comorbidities.^2^, ^12^, ^13^, ^14^, ^15^ Moreover, a Chinese single‐center study^16^ published in 2015 found that persistent or permanent AF was more closely related to thromboembolism than paroxysmal AF (HR: 2.81, 95% CI: 1.25–6.32, *p =* .012). Recently data from 5 AF registries in Japan also indicated that NPAF increased the risk of ischemic stroke after adjusting for OAC administration at enrollment (HR, 1.59; 95% CI, 1.21–2.10; *p =* .001).^17^ And the analysis of the Chinese atrial fibrillation registry,^18^ which included non‐valvular AF patients enrolled between August 2011 and June 2015, reported an increased risk of stroke, all‐cause death in non‐anticoagulated NPAF patients compared with PAF group, but same between anticoagulated PAF and NPAF patients. However, after adjustment, AF type was not an independent predictor of thromboembolism in NVAF patients. Similar results were presented in the subanalysis of the J‐RHYTHM Registry.^19^ Besides, EORP‐AF General Pilot Registry^11^ reported comparable risk between NPAF and PAF under the high rates of anticoagulation use. Two reasons might explain the controversial results. On the one hand, the AF type might change during the follow‐up. Furthermore, AF progression significantly increased the risk of adverse events,^20^, ^21^, ^22^ especially during the progression period from paroxysmal AF to sustain AF.^23^ The Chinese atrial fibrillation registry had a follow‐up period of up to 4 years. Previous study^23^ reported that the cumulative rate of progression to sustained type was only 2.6% at 1 year, 11.4% at 3 years, and 28.3% at 5 years, which indicated that the progression of AF type was time‐dependent. Our study only had a 1‐year follow up, which relatively reduced the confounding effects due to AF progression. On the other hand, anticoagulated therapy might diminish the difference in thromboembolic risk resulting from the AF pattern. According to the Chinese atrial fibrillation registry,^18^ Stockholm cohort trial^13^ and the ACTIVE W study,^24^ NPAF significantly increased the risk of TE events in the subgroup of patients without OAC, though the significance was not seen in the whole population. Both the EORP‐AF General Pilot Registry^11^ and the subanalysis of the J‐RHYTHM Registry^19^ in which no difference in TE events was found between types, both had a high proportion of anticoagulation therapy. In our study, anticoagulated status during follow‐up was collected, and patients initiating OAC during the follow‐up were excluded, which minimized its effect on the results. In addition to higher risk of stroke, stroke due to NPAF are associated with a worse acute clinical course and greater volume of infarction than those due to PAF.^25^

The role of AF types in the subgroups was also explored. Regarding thromboembolism, the results were consistent in all subgroups. As for the all‐cause death, the trend preserved in all subgroups except for the patients discharged without antiplatelet agents. The limited sample size might contribute to the difference. Further, considering the admission classification defined by physician's interpretation might be subjective, we performed a sensitivity analysis using a relatively objective classification according to the electrocardiogram rhythm at discharge. The main findings kept consistent. All results proved that NPAF was still an independent predictor of adverse outcomes, even after adjusting for the previously reported risk factors.

We also evaluated the potential of the AF pattern in decision‐making for stroke prevention. Though increasing evidence showed that patterns or duration of AF associated with adverse events,^26^, ^27^ it is essential whether the difference due to AF pattern could affect our daily clinical practice. Recently, several papers have shown a reduction in all‐cause mortality, hospitalization for heart failure or the composite endpoint of adverse outcomes with AF catheter ablation in patients with reduced ejection fraction.^28^, ^29^, ^30^ Furthermore, the newest published results of EAST‐AFNET 4 Trial^31^ demonstrated that early rhythm control based on the usual care for AF could reduce the incidence of adverse outcomes including cardiovascular death, stroke, hospitalization for heart failure and acute coronary syndrome(HR 0.79, 95%CI 0.66–0.94, *p =* .005). The literature suggested that reducing the burden of AF can improve prognosis. The statistical difference on the risk of TE events was reached in the post hoc analysis of the large clinical trials,^14^, ^32^, ^33^ but the results had no impact on the clinical decision‐making in patients who were on anticoagulation. Moreover, the yearly event rate of thromboembolism in the PAF group was still high in patients with novel OACs (1.73% in the ROCKET‐AF trial^32^; 1.49% in the ENGAGE AF‐TIMI 48 trial^33^; 0.98% in the ARISTOTLE trial^14^; about 1.0% in the RE‐LY study^34^). Manolis et al^35^ thought that with regards to those with a CHA_2_DS_2_‐VASc score of 0, one might wish to consider additional risk factors (including NPAF or AF burden) beyond those in scores to decide whether there was a need for thromboembolic protection that outweighs the bleeding risk. The present study assessed the role of NPAF in patients without OAC and evaluated its value by adding it to the CHA_2_DS_2_‐VASc score, and an improvement was seen. The arbitrary classification of AF type might limit the assistance for improving the predictive utility of the CHA_2_DS_2_‐VASc score on TE events. While last year investigators reported^36^ that NPAF and renal dysfunction were strongly associated with left atrial thrombus, and the area under the curve for predicting left atrial thrombus significantly increased to 0.81 after cooperating the two predictors into CHA_2_DS_2_‐VASc score. NPAF was assigned a weight of 4 or 10 points in that paper, yet it was only 1 point in our study. In that case, the AF pattern might be used to identify patients who needed anticoagulation. Meanwhile, the weight of the AF pattern in the risk prediction model needs more investigated.

There were several limitations in the present study. First, the database did not consist of all known risk factors related to TE events, such as left atrial diameter, high‐sensitivity troponin, N‐terminal pro‐B‐type natriuretic peptide. We cannot rule out the possibility of unmeasured or residual confounding. Second, the relatively small sample size might lead to our exaggerative estimation of the association between NPAF and thromboembolic risk. Nevertheless, a similar trend was previously reported. Third, the diagnosis of the type of AF was physician‐defined and arbitrary, and the distinction between paroxysmal and persistent AF is often not made correctly without access to long‐term monitoring. Considering that, we performed a sensitivity analysis by classifying the patients according to the electrocardiogram at discharge to explore the association. And the results were consistent. Fourth, the AF pattern might progress from paroxysmal to persistent or permanent AF,^23^ and risk factor profile might change,^37^, ^38^ which both associated with stroke and might be confounders in our study. Nevertheless, the follow‐up period was only 1 year in the present study. In that condition, the changes might have a limited effect on our results. Fifth, there was no external cohort to validate the utility after adding NPAF into the CHA_2_DS_2_‐VASc score. Our results only propose the hypothesis that adding NPAF in the CHA_2_DS_2_‐VASc score could improve the prediction accuracy for thromboembolism in AF patients. Further studies with larger sample size were needed to validate the results and investigate how much weight should be given to NPAF when integrating it into the existing risk prediction model. In addition, the present study could not accurately assess the AF burden. Further studies based on remote monitoring devices are needed.

## 5 CONCLUSIONS

In Chinese non‐anticoagulated AF patients, NPAF was an independent predictor of thromboembolism and mortality. The addition of NPAF to the CHA_2_DS_2_‐VASc score allowed an improvement in the accuracy of the prediction of thromboembolic events. Further investigations are needed to confirm the results and assess its utility in therapy strategy.

## CONFLICT OF INTEREST

The authors declare no potential conflict of interest.

## Supporting information


**Figure S1** Flowchart of the study patients.

**Table S1** Baseline characteristics of patients divided by electrocardiogram at discharge.

**Table S2** The risk of outcomes in patients discharged with atrial fibrillation/flutter comparing to sinus rhythm.

**Table S3** The baseline risk factors of thromboembolic events.

## Data Availability

The data that support the findings of this study are available from the corresponding author upon reasonable request.
