# Supplementary figures and images for: Type of atrial fibrillation and outcomes in patients without oral anticoagulants
Source: Clin Cardiol. 2020 Dec 12;44(2):168–75. doi: 10.1002/clc.23519 (PMC7852164; doi:10.1002/clc.23519)

Figure S1. Flowchart of the Study Patients.

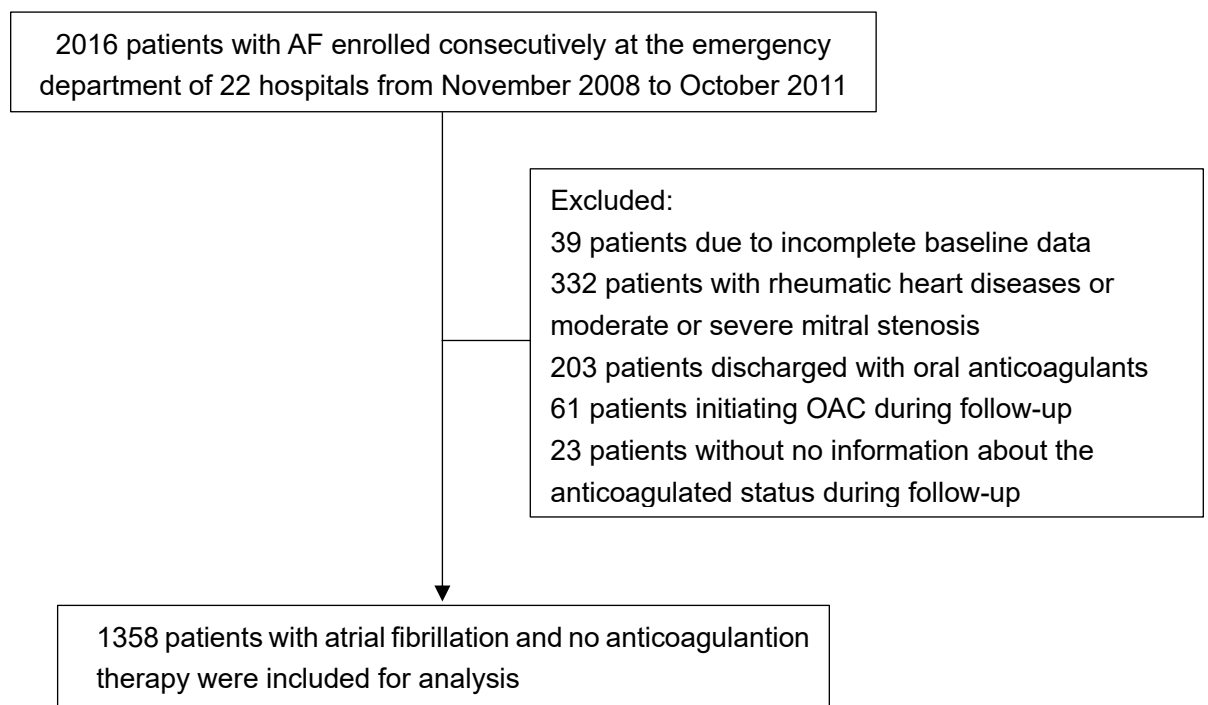

Supplement: Supplementary file 1 — Figure S1 Flowchart of the study patients. [file CLC-44-168-s001.pdf]
