# Supplementary material for: Type of atrial fibrillation and outcomes in patients without oral anticoagulants
Source: Clin Cardiol. 2020 Dec 12;44(2):168–75. doi: 10.1002/clc.23519 (PMC7852164; doi:10.1002/clc.23519)
Supplement: Supplementary file 2 — Table S1 Baseline characteristics of patients divided by electrocardiogram at discharge. [file CLC-44-168-s002.docx]

**Table S1. Baseline characteristics of patients divided by electrocardiogram at discharge.**

| **Variables** | **Other rhythm** | **Atrial fibrillation/flutter** | **P-value** |
| --- | --- | --- | --- |
|  | **n=375, 27.6%** | **n=983, 72.4%** |  |
| **Demographics** |  |  |  |
| Female,n(%) | 205(54.7%) | 477(48.5%) | 0.043 |
| Age,years | 67.8±13.9 | 72.1±11.9 | <0.001 |
| BMI,kg/m^2^ | 24.3±3.9 | 23.6±3.7 | 0.032 |
| SBP at discharge,mmHg | 133±22.9 | 135±23 | 0.167 |
| DBP at discharge,mmHg | 79.8±13.6 | 81.1±15.3 | 0.199 |
| HR at discharge,bpm | 111±33.5 | 100.5±27.6 | <0.001 |
| CHA_2_DS_2_-VAScs score | 3.2±2.0 | 3.9±2.0 | <0.001 |
| Tobacco use,n(%) | 71(18.9%) | 249(25.3%) | 0.013 |
|  |  |  |  |
| **Medical history,n(%)** |  |  |  |
| Hypertension | 239(63.7%) | 607(61.7%) | 0.5 |
| Heart failure | 58(15.5%) | 405(41.2%) | <0.001 |
| Coronary artery disease | 138(36.8%) | 545(55.4%) | <0.001 |
| Previous myocardial infarction | 24(6.4%) | 92(9.4%) | 0.081 |
| Previous stroke or TIA | 62(16.5%) | 194(19.7%) | 0.177 |
| Diabetes mellitus | 49(13.1%) | 183(18.6%) | 0.015 |
| Significant valvular heart disease | 2(0.5%) | 44(4.5%) | <0.001 |
| Emphysema/COPD | 28(7.5%) | 152(15.5%) | <0.001 |
| Hyperthyroidism | 15(4%) | 38(3.9%) | 0.909 |
| Sleep apnea | 10(2.7%) | 44(4.5%) | 0.127 |
| Major bleeding | 5(1.3%) | 28(2.8%) | 0.105 |
| Dementia or cognitive defects | 4(1.1%) | 29(3%) | 0.044 |
| Prior AF catheter, surgical ablation or Maze procedure | 2(0.5%) | 9(0.9%) | 0.649 |
|  |  |  |  |
| **Medication at discharge,n(%)** |  |  |  |
| Antiplatelet drug | 211(56.3%) | 657(66.8%) | <0.001 |
| β-blocker | 150(40%) | 445(45.3%) | 0.08 |
| ACEI/ARB | 131(34.9%) | 410(41.7%) | 0.023 |
| Calcium channel blocker | 119(31.7%) | 249(25.3%) | 0.018 |
| Diuretics | 53(14.1%) | 389(39.6%) | <0.001 |
| Digoxin | 34(9.1%) | 268(27.3%) | <0.001 |
| Statin | 105(28%) | 297(30.2%) | 0.424 |
| Antiarrhythmic drug | 63(16.8%) | 101(10.3%) | 0.001 |

BMI: Body mass index; SBP: Systolic blood pressure; DBP: Diastolic blood pressure; HR: Heart rate; TIA: Transient ischemic attack; COPD: Chronic obstructive pulmonary disease; AF: Atrial fibrillation; ACEI/ARB: Angiotensin-converting enzyme inhibitor/angiotensin receptor antagonist.
