# Supplementary material for: Type of atrial fibrillation and outcomes in patients without oral anticoagulants
Source: Clin Cardiol. 2020 Dec 12;44(2):168–75. doi: 10.1002/clc.23519 (PMC7852164; doi:10.1002/clc.23519)
Supplement: Supplementary file 3 — Table S2 The risk of outcomes in patients discharged with atrial fibrillation/flutter comparing to sinus rhythm. [file CLC-44-168-s003.docx]

Table S2. The risk of outcomes in patients discharged with atrial fibrillation/flutter comparing to sinus rhythm.

|  | Numbers of events (yearly rate,%) | | |  | Univariable analysis | | |  | Multivariable analysis^a^ | |
| --- | --- | --- | --- | --- | --- | --- | --- | --- | --- | --- |
| Outcomes | All patients | Patients with other rhythm | Patients with atrial fibrillation/flutter |  | HR(95%CI) |  | P-value |  | HR(95%CI) | P-value |
| Thromboembolism | 107(8.1) | 17(4.5) | 90(10.2) |  | 2.213(1.318-3.717) | | 0.003 |  | 2.502(1.451-4.314) | 0.001 |
| Stroke | 101(7.7) | 16(4.3) | 85(9.6) |  | 2.218(1.3-3.783) | | 0.003 |  | 2.544(1.452-4.457) | 0.001 |
| All-cause death | 215(16.4) | 35(9.2) | 180(19.3) |  | 2.074(1.444-2.978) | | <0.001 |  | 1.729(1.174-2.547) | 0.006 |
| Cardiovascular death | 122(9.3) | 14(3.7) | 108(11.6) |  | 3.12(1.788-5.444) | | <0.001 |  | 2.08(1.151-3.759) | 0.015 |

CI, confidence interval; HR, hazard ratio.

^a^Adjusted for sex, age ≥75 years old, body mass index, admission systolic blood pressure, admission diastolic blood pressure, admission heart rate, tobacco use, previous stroke or transient ischemic attack, coronary artery diseases, previous myocardial infarction, hypertension, heart failure, significant valvular heart disease, diabetes mellitus, emphysema/ chronic obstructive pulmonary disease, hyperthyroidism, sleep apnea, previous major bleeding, dementia or cognitive defects, antiplatelet drug, β-blocker, ACEI/ARB, calcium channel blocker, diuretics, digoxin, statin, antiarrhythmic drug.
