# Supplementary material for: Type of atrial fibrillation and outcomes in patients without oral anticoagulants
Source: Clin Cardiol. 2020 Dec 12;44(2):168–75. doi: 10.1002/clc.23519 (PMC7852164; doi:10.1002/clc.23519)
Supplement: Supplementary file 4 — Table S3 The baseline risk factors of thromboembolic events. [file CLC-44-168-s004.docx]

Table S3. The baseline risk factors of thromboembolic events.

|  | Univariate analysis | | Multivariate analysis^*^ | |
| --- | --- | --- | --- | --- |
| Risk factor | P-value | HR(95%CI) | P-value | HR(95%CI) |
| Age ≥ 75 years | **<0.001** | 2.29(1.55-3.38) | **0.002** | 1.94(1.26-2.99) |
| Female | 0.054 | 1.46(0.99-2.15) | **0.034** | 1.64(1.04-2.59) |
| Prior stroke or TIA | **<0.001** | 2.42(1.62-3.61) | **0.018** | 1.7(1.1-2.64) |
| Hypertension | **0.018** | 1.67(1.09-2.56) | 0.176 | 1.43(0.85-2.4) |
| Diabetes | 0.22 | 1.34(0.84-2.14) | 0.929 | 0.98(0.6-1.6) |
| Heart failure | 0.147 | 0.73(0.47-1.12) | 0.13 | 0.68(0.41-1.12) |
| Coronary artery disease | 0.666 | 1.09(0.74-1.59) | 0.293 | 0.79(0.5-1.23) |

The P-value is from the Wald test, Cox proportional hazards model

^*^: Adjusted for sex, age ≥75 years old, body mass index, admission systolic blood pressure, admission diastolic blood pressure, admission heart rate, tobacco use, previous stroke or transient ischemic attack, coronary artery diseases, previous myocardial infarction, hypertension, heart failure, significant valvular heart disease, diabetes mellitus, emphysema/ chronic obstructive pulmonary disease, hyperthyroidism, sleep apnea, previous major bleeding, dementia or cognitive defects, antiplatelet drug, β-blocker, ACEI/ARB, calcium channel blocker, diuretics, digoxin, statin, antiarrhythmic drug.
